# Uncovering the Antifungal Potential of Plant-Associated Cultivable Bacteria from the Aral Sea Region against Phytopathogenic Fungi

**DOI:** 10.3390/pathogens13070585

**Published:** 2024-07-15

**Authors:** Ilkham S. Aytenov, Tohir A. Bozorov, Daoyuan Zhang, Sitora A. Samadiy, Dono A. Muhammadova, Marufbek Z. Isokulov, Sojida M. Murodova, Ozoda R. Zakirova, Bakhodir Kh. Chinikulov, Anvar G. Sherimbetov

**Affiliations:** 1 Key Laboratory of Ecological Safety and Sustainable Development in Arid Lands, Xinjiang Key Laboratory of Conservation and Utilization of Plant Gene Resources, Xinjiang Institute of Ecology and Geography, Chinese Academy of Sciences, Urumqi 830011, China; ilhamaytenov@gmail.com (I.S.A.); zhangdy@ms.xjb.ac.cn (D.Z.); 2 Laboratory of Molecular and Biochemical Genetics, Institute of Genetics and Plants Experimental Biology, Uzbek Academy of Sciences, Kibray 111226, Uzbekistan; sitorasamadiy@gmail.com (S.A.S.); donoxyz91@gmail.com (D.A.M.); murodovasojida1433@gmail.com (S.M.M.);; 3 Department of Microbiology and Biotechnology, National University of Uzbekistan, University Street, 4, Tashkent 100174, Uzbekistan; 4 Laboratory of Plant Immunity, Institute of Genetics and Plants Experimental Biology, Uzbek Academy of Sciences, Kibray 111226, Uzbekistan

**Keywords:** antagonistic bacteria, antifungal activity, Aral Sea, enzymatic activities, salt tolerance

## Abstract

Two freshwater rivers, the Amu Darya and Syr Darya, flow into the Aral Sea, but they began to diminish in the early 1960s, and by the 1980s, the lake had nearly ceased to exist due to excessive water consumption for agriculture and the unsustainable management of water resources from rivers, which transformed the Aral Sea into a hypersaline lake. Despite this, the flora and fauna of the region began to evolve in the high-salinity seabed soil, which has received little attention in studies. In this study, we isolated approximately 1400 bacterial strains from the rhizosphere and phyllosphere of plant species of distinct families. Bacterial isolates were examined for antifungal activities against a range of pathogenic fungi such as *Rhizoctonia gossypii*, *Trichothecium ovalisporum*, *Fusarium annulatum*, *F. oxysporum*, *F. culmorum*, *F. brachygibbosum*, *F. tricinctum*, *F. verticillioides*, *Alternaria alternata*, *A. terreus*, *Aspergillus niger*, and *As. flavus*. Eighty-eight bacterial isolates exhibited varying antagonistic ability against pathogenic fungi. Furthermore, DNA barcoding of isolates using the 16S rRNA gene indicated that most antagonistic bacteria belonged to the *Bacillus* and *Pseudomonas* genera. The study also explored the activity of hydrolytic and cell-wall-degrading enzymes produced by antagonistic bacteria. The findings revealed that antagonistic bacteria can be utilized to widely protect seabed plants and plants growing in saline areas against pathogenic fungi, as well as agricultural crops.

## 1. Introduction

The Aral Sea, once one of the world’s largest inland bodies of water, has experienced a dramatic reduction in size over the past five decades. This decline is primarily due to human activities such as extensive irrigation projects diverting water from the two main rivers that feed the sea, the Amu Darya and the Syr Darya, for agricultural purposes. As a result, the inflow of water into the Aral Sea has significantly decreased, leading to its shrinking [1,2]. The formation of a saline layer at the bottom of the sea [3] due to reduced water levels has led to the emergence of significant saline storms in the lands of Central Asia [4]. Soil salinization is indeed a widespread issue that affects agricultural productivity in various regions globally; as a result, soil salinization can lead to food shortages, economic losses for farmers, and environmental degradation [5]. Studies demonstrated that salt stress can indeed increase the susceptibility of plants to various phytopathogens. This weakened state makes plants more vulnerable to pathogen invasion, thus negatively impacting crop growth and production [6,7].

Plant diseases are indeed a major concern for global agriculture, with significant implications for food security and economic stability. The estimate that approximately 40% of the world’s major crops are lost annually due to plant diseases underscores the scale of this problem [8,9]. Plant diseases not only cause reductions in crop productivity but also have detrimental effects on crop quality. Many plant pathogens have the ability to survive in soil as dormant structures for extended periods of time, sometimes even for years, and serve as a reservoir of infection potential, allowing pathogens to persist in the absence of a suitable host plant [10,11,12,13]. Diseases caused by soil-borne plant pathogens are indeed among the most serious challenges in agriculture and can cause significant damage to crops by infecting plant roots, stems, and other below-ground plant parts and making it difficult to manage them [14,15]. Chemical fungicides are currently necessary for effective disease control. In recent decades, the control of soil-borne diseases has mostly depended on chemical pesticides and played a major role in pest management, resulting in preserving and/or improving production [16]. The long-term, intensive use of pesticides in agriculture has negative consequences, including the enhancement of pathogenic fungi resistance, environmental pollution, adverse effects on human health, and an ever-increasing cost of production [17,18]. 

The use of biofertilizers and biopesticides in agriculture is gaining momentum globally due to several factors, including food safety concerns, environmental sustainability, and the need to reduce reliance on synthetic chemicals [19,20]. Soil salinity is a significant issue affecting agricultural lands worldwide, with it estimated that more than 1 billion hectares of soil globally exhibit varying degrees of salinity [21]. Halophytes, plants that can thrive in saline environments, have evolved various strategies to cope with abiotic and biotic stressors, often with the assistance of their associated microbiomes [8]. Biological control offers a sustainable and environmentally friendly alternative to chemical pesticides for managing plant diseases and pests. Many biological control agents have been discovered through screening large numbers of soil or plant-associated microorganisms for their ability to suppress phytopathogens either in vitro or in planta [22,23,24,25]. The rhizosphere, the soil environment directly surrounding plant roots, is known to harbor a diverse and dynamic community of microorganisms, including bacteria with antagonistic activity against plant pathogens, and Bacilli and Pseudomonads are indeed among the most widespread bacterial isolates found in the rhizospheres of plants [22,23,26,27]. *Bacillus*, *Pseudomonas*, and *Streptomyces* species are among the most commonly used bacterial genera for the biocontrol of phytopathogenic fungi [28]. 

Studying microbial diversity in stressed environments, such as hypersaline seabeds like those found in the diminished Aral Sea, is crucial for understanding ecological interactions between microorganisms and their hosts in stressed environments that can provide valuable insights into host survival and adaptation strategies. The investigation of bacteria that are particularly beneficial for agriculture is more important than ever. Culture-independent bacterial identifications from hypersaline conditions, such as those found in salt lakes, saline soils, or hypersaline brines, consistently reveal a remarkably high microbial diversity and abundance of uncharacterized halophilic microbes [29]. In hypersaline conditions, the most common bacterial phyla that have been detected include Proteobacteria, Bacteroidetes, Firmicutes, Actinobacteria, Deinococcus-Thermus, and Verrucomicrobia [30]. Despite the considerable attention paid to the environmental challenges facing the Aral Sea, including its shrinking and the resulting ecological impacts, the diversity of bacteria in this region, particularly antagonistic bacteria, remains relatively understudied [31,32,33]. Therefore, it is important to study the diversity of bacteria in the diminished seabed of Aral. 

In this work, we examined the antagonistic ability of numerous bacteria associated with plants growing on the southwestern and southern Aral Sea seafloor against several pathogenic fungi. Our research aims were (1) to isolate bacteria from the plant rhizosphere and phyllosphere; (2) to determine their antagonistic properties against phytopathogenic fungi; (3) to perform molecular identification of antagonistic bacteria; (4) to determine their salt tolerance; and (5) to measure their enzymatic activities. The increasing salinity of agricultural lands presents significant challenges for farmers, affecting crop productivity and making it difficult to manage plant diseases caused by pathogenic fungi. Employing beneficial bacterial antagonists as fungicides offers a promising solution to address these challenges.

## 2. Materials and Methods 

### 2.1. Cite Description, Plant Samples, and Fungal Strains

Plant samples were collected from the southern and western parts of the Aral Sea. The GPS coordinates and elevation were recorded. This region is located at the transition point between the temperate (subboreal) and subtropical desert zones of Uzbekistan. It is known for its sharply continental climate, which is characterized by very low precipitation (87–108 mm annually), hot summer temperatures (up to 42 °C), and low winter temperatures (as low as −31 °C) [34].

Plants from the Lamiaceae, Poaceae, Chenopodiaceae, Boraginaceae, Tamaricaceae, Euphorbiaceae, Solanaceae, Apiaceae, Zygophyllaceae, Asteraceae, Polygonaceae, and Fabaceae families that are common in that region were collected. Plant samples were collected into sterile bags, transported to the laboratory, and kept at 4 °C for future use.

Several phytopathogenic fungi, such as *Rhizoctonia gossypii*, *Trichothecium ovalisporum*, *Fusarium annulatum*, *F. oxysporum*, *F. culmorum*, *F. brachygibbosum*, *F. tricinctum*, *F. verticillioides*, *Alternaria alternata* (IGPEB-1, IGPEB-2), *A. terreus*, *Aspergilus niger*, and *As. flavus*, were obtained from “the Unique collections of phytoopathogs and other microrganisms” of the Institute of Genetics and Plant Experimental Biology of the Academy of Uzbekistan.

### 2.2. Bacterial Isolation 

The collected plant samples were homogenized, and 1 mL of sterilized PBS buffer (137 mM NaCl, 2.7 mM KCl, 1 mM Na_2_HPO_4_, and 1.8 mM KH_2_PO_4_; pH 7.4) was added and mixed. The solution was serially diluted up to 10^−6^ with sterile buffer. Each diluted sample was placed over nutrient agar (NA) (0.5% peptone, 0.3% beef extract, 1.5% agar, pH 6.8) (Difco, France) in a laminar flow cabinet. The plates were placed in a thermostatic incubator at 28 degrees Celsius for 48–96 h until bacterial colonies appeared. The morphology of the bacterial colonies on agar was distinct, including form, size, margin, and elevation.

### 2.3. Isolation of Antagonistic Bacteria

To isolate antagonistic bacteria against phytopathogenic fungi, half NA medium and half potato dextrose agar (PDA) (potato starch 4 g L^−1^, dextrose 20 g L^−1^, and agar 15 g L^−1^, pH 7.2) mixed medium was used. A piece of gel with a diameter of 1 cm from a PDA plate with a pure fungal culture was transferred onto the center of the mixed medium. Four bacterial isolates were cultivated at a distance of 2 mm from the fungal gel in the center. The study estimated the inhibition of fungal mycelial growth by antagonistic bacteria by calculating the distance between the bacterial growth edge and the fungi growth edge, using the formula described by Alenezi et al. [35]:

I (%) = (1 − a/b) × 100

where ‘a’ is the distance between the center of the fungal colony and the growing edge on the bacterial side and ‘b’ is the fungal colony’s radius of control.

### 2.4. Molecular Identification

Bacterial genomic DNA was isolated using the standard CTAB method. The 16S rRNA gene was amplified using primer pairs 27F (5′-AGAGTTTGATCATGGCTCAG-3′) and 1492R (5′-TACGGCTACCTTGTTACGACTT-3′) [36]. The PCR conditions were as follows: 10 min at 95 °C for the initial denaturation step, followed by 35 cycles (denaturation at 94 °C for 10 s, annealing at 55 °C for 30 s, elongation at 72 °C for 2 min), and a final extension at 72 °C for 10 min. The PCR products were visualized on a 1.5% agarose gel. PCR products were purified and sequenced bidirectionally with the Sanger method at Sangon Biotech (Shanghai, China).

### 2.5. Sequence Analysis

Sequence assembly and analysis were performed using SeqMan software from the DNASTAR Lasergene 7 (V. 7.2.1) package. The bacterial 16S RNA sequences were compared to the publicly accessible bacterial species in GenBank using the BLASTN algorithm. Sequences with high identity were rated. These sequences were further matched using CLUSTALW in MEGA11. Finally, a maximum likelihood (ML) phylogenetic tree was generated in MEGA11 using the neighbor-joining algorithm based on the Tajima–Nei model, with 5000 bootstrap iterations.

### 2.6. Determination of Enzymatic Activities

Bacterial protease activity was evaluated on an agar plate (10 g L^−1^ casein, 1 g L^−1^ glucose, 1 g L^−1^ yeast extract, 1 g L^−1^ K^2^HPO^4^, 0.5 g L^−1^ KH^2^PO^4^, 0.1 g L^−1^ MgSO^4^, 20 g L^−1^ agar, pH 7.0). Bacterial isolates were grown on casein plate agar and incubated at 30 °C for 72 h. Protease activity was visualized by the creation of distinct halos surrounding the colony, indicating that the bacteria hydrolyzed the proteins [37,38].

To test the bacteria’s lipolytic activity, nutrient agar was supplemented with 10 mL—1 Tween 85. Cool-filtered Tween 85 was added to the pre-cooled medium and carefully mixed. Bacteria are cultured 30 °C for 96 h. Clear halos developed surrounding the bacterial colony, suggesting lipolytic activity [38,39].

Lygnocellulotic activities such as cellulotic, xylanase, cellobiase, and glucanase activities were measured by utilizing relevant substrates such as carboxymethylcellulose, 4-nitrophenyl beta-D-xylopyranoside, 4-nitrophenyl beta-D-glucopyranoside, and 4-nitrophenyl beta-D-cellobiose [40,41,42].

### 2.7. Bacterial Salt Tolerance Assay 

The resistance of the identified antagonistic bacterial strains to salt was assessed by examining their growth on NA media with various concentrations of NaCl (2.5%, 5%, 7.5%, 10%, and 15%). The plates were incubated at 28 ± 2 °C for 48–96 h. The growth capacity of bacterial colonies in the media was measured.

## 3. Results

### 3.1. Research Site and Plant Collection

**Figure 1:**
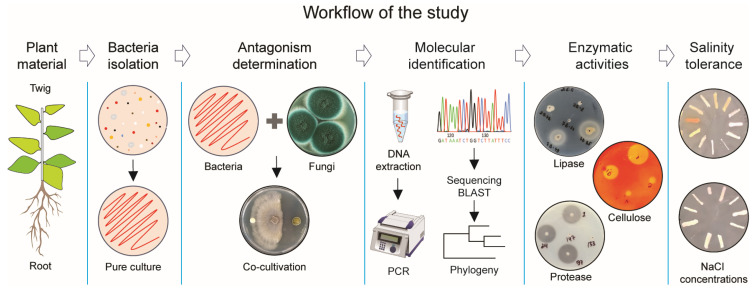
Research strategy to identify bacterial antagonists against pathogenic fungi.

To determine the antifungal activity of bacteria associated with plants grown in an area of the Aral Sea capable of inhibiting phytopathogenic fungi, including their enzymatic activities and salt tolerance ability, we developed an exploratory workflow for the stepwise identification of bacterial isolates, as illustrated in Figure 1.

**Figure 2:**
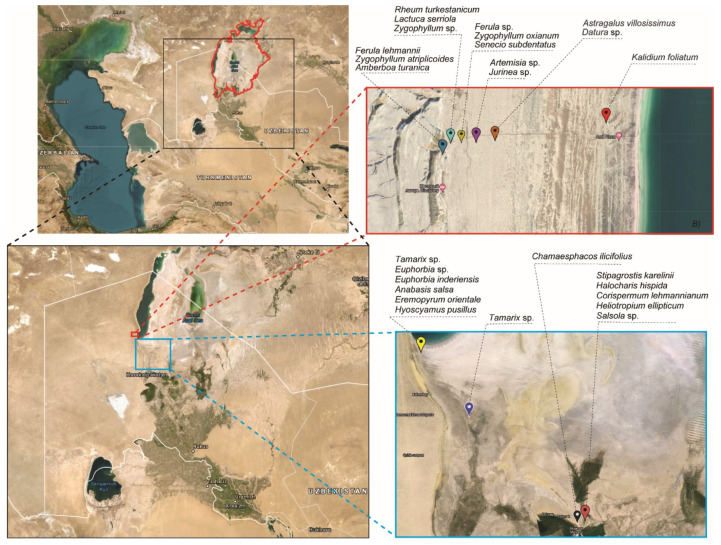
Maps of the Aral sea and of sample collecting sites. The red bold line on the map depicts the lake’s initial boundary in 196-, before it shrank. The dashed red line represents the location of samples from the western Aral Sea, while the blue dashed line represents sample collection from the lake’s central and southern seabeds. Black dashed lines indicate the names of collected plant species.

The investigation was carried out on the Aral Sea’s seabed, mostly along the southern and western coasts (Table 1; Figure 2), since gradually decreased sea water positively correlates with the level of salinity in soil. Twenty-seven unique plant species were collected from those locations.

### 3.2. Identification of Cultivable Wild Plant-Associated Bacteria

Research was carried out to assess the antagonistic activity of 1400 isolated bacteria against 12 distinct pathogenic fungal species. Isolates were collected from the rhizosphere and phyllosphere of plants of various species. Only 88 of the 1400 bacterial isolates showed antagonistic activity against various pathogenic fungi. A total of 88 antagonistic isolates were analyzed using 16S rRNA gene sequencing for molecular identification. The molecularly identified OTUs were compared to publicly accessible sequences in GenBank using the BLASTN algorithm search. The analysis identified 15 operational taxonomic units (OTUs), which were distributed across three phyla: Firmicutes, Pseudomonadota, and Actinomycetota. Furthermore, the isolates were classified into four classes—Bacilli, Gammaproteobacteria, Alphaproteobacteria, and Actinomycetes—and five orders—Bacillales, Pseudomonadales, Hyphomicrobiales, Kitasatosporales, and Micrococcales. The isolates were also sorted into six families, including Bacillaceae, Paenibacillaceae, Pseudomonadaceae, Phyllobacteriaceae, Micrococcaceae, and Streptomycetaceae, and seven genera—*Bacillus*, *Paenibacillus*, *Peribacillus*, *Pseudomonas*, *Phyllobacterium*, *Kocuria*, and *Streptomyces* (Table 2).

**Figure 3:**
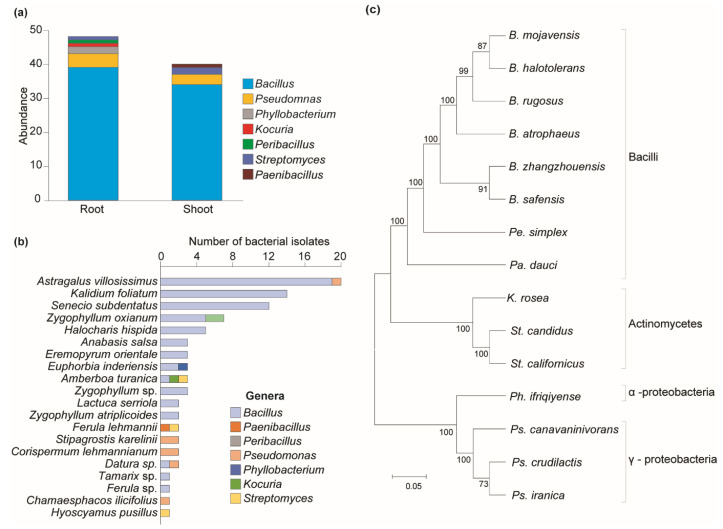
Distribution of bacterial diversity across different plant parts and their phylogenetic relationships. (**a**) Distribution of bacterial genera by plant parts. (**b**) Distribution of antagonistic bacteria among plant species. (**c**) Clustering analysis of antagonistic bacteria. Maximum likelihood was inferred using the neighbor-joining method. The percentage of replicate trees in which the associated taxa clustered together in the bootstrap test (1—replicates) is shown next to the branches. The evolutionary distances were computed using the Tajima–Nei method and are in units of the number of base substitutions per site. The distance scale represents the number of differences between the sequences. Evolutionary analyses were conducted in MEGA 11.

Among antagonistic bacteria, 48 isolates were obtained from the rhizosphere and 40 from the phyllosphere parts of plants (Figure 3a). Most antagonistic bacteria found in both plant parts belonged to the *Bacillus* genus, which was mainly found in 15 plant species out of 20 (Figure 3b). *Pseudomonas* species were found in five plant species. Bacillota was the biggest phylum (86%), followed by Proteobacteria and Actinomycetota. Bacilli was the biggest class, while Gammaproteobacteria was the largest within Proteobacteria (Figure 3c).

This study found that 41 isolates of antagonistic bacteria were obtained only from the rhizosphere parts of nine plants (*Ferula* sp.—1, *Lactuca serriola*—2, *Senecio subdentatus*—12, *Kalidium foliatum*—14, *Chamaesphacos ilicifolius*—1, *Stipagrostis karelinii*—2, *Zygophyllum atriplicoides*—2, *Zygophyllum oxianum*—7). Thirty-six isolates were obtained only from the phyllosphere part of seven plants (*Ferula lehmannii*—2, *Halocharis hispida*—5, *Astragalus villosissimus*—20, *Eremopyrum orientale*—3, *Hyoscyamus pusillus*—1, *Datura* sp.—1, *Tamarix* sp.—1, *Zygophyllum* sp.—3), and the remaining eleven isolates were obtained from both the rhizosphere and twig parts of four plants (*Amberboa turanica*—3, *Corispermum lehmannianum*—2, *Anabasis salsa*—3, *Euphorbia inderiensis*—3). No isolates of bacteria with antagonistic ability were detected from *Artemisia* sp., *Jurinea* sp., *Heliotropium ellipticum*, *Salsola* sp., *Euphorbia* sp., *Rheum turkestanicum*, or *Tamarix* sp. (Table 3).

### 3.3. Antifungal Activity of Bacterial Isolates against Phytopathogenic Fungi

**Figure 4:**
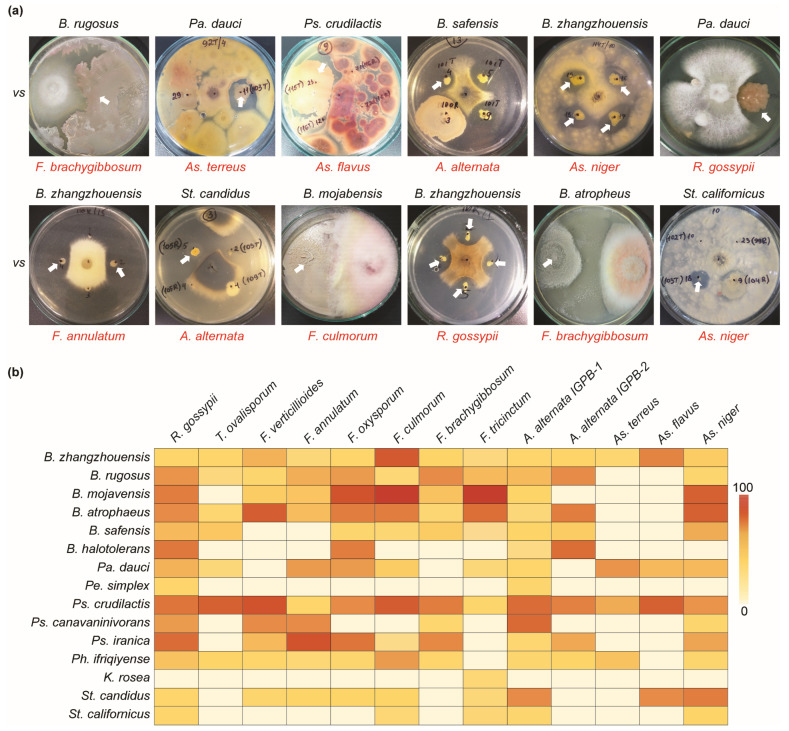
Antifungal activity of bacterial isolates against phytopathogenic fungi. (**a**) Examples of the antifungal abilities of different antagonistic bacteria. The name in red indicates phytopathogenic fungi. (**b**) Heatmap depicting the antifungal activities of antagonistic bacterial species. The zero-to-one-hundred scale shows the percentage of bacterial antifungal ability.

The co-cultivation method in a dual PDA/NA (50%/50%) medium was used to determine the antagonistic ability of all bacterial isolates against pathogenic *Rhizoctonia gossypii*, *Trichothecium ovalisporum*, *Fusarium annulatum*, *F. oxysporum*, *F. culmorum*, *F. brachygibbosum*, *F. tricinctum*, *F. verticillioides*, *Alternaria alternata* (IGPEB-1, IGPEB-2), *A. terreus*, *As. niger*, and *As. flavus*. The use of a dual medium enabled the simultaneous cultivation of fungus and bacteria in a mixed medium (Figure 4). Mainly, all 1400 bacterial isolates from the plant rhizosphere and phyllosphere were examined against several phytopathogenic fungi. The result revealed that 88 bacteria out of 1400 bacterial isolates had antifungal activity against one or more phytopathogenic fungi with varying degrees of fungal mycelial growth inhibition (Figure 4a). Among antagonistic bacteria, *B. zhangzhouensis* and *Ps. crudilactis* showed antagonistic abilities against all thirteen pathogenic fungi, with varying inhibitory activities (Figure 4b). *Bacillus rugosus*, *B. atrophaeus*, and *Ph. ifriqiyense* were able to inhibit eleven fungi, while *Pa. dauci* inhibited ten pathogenic fungi. *Bacillus mojavensis*, *B. safensis*, *Ps. iranica*, and *St. candidus* showed the inhibition of nine pathogenic fungi. Furthermore, *K. rosea* and *Pe. simplex* showed the least antifungal activity. Among pathogenic fungi, *R. gossypii*, *A. alternata* IGBEP-1, and *As. niger* were the most susceptible to antagonistic bacteria. The fewest bacterial isolates showed antagonistic properties against *As. flavus* (3) and *As. terreus* (4). *Bacillus zhangzhouensis* was the most common antagonistic bacterium (Table 2 and Table 3), with an estimated 47 isolates.

### 3.4. Determination of the Enzymatic Activities of the Antagonistic Bacteria

**Figure 5:**
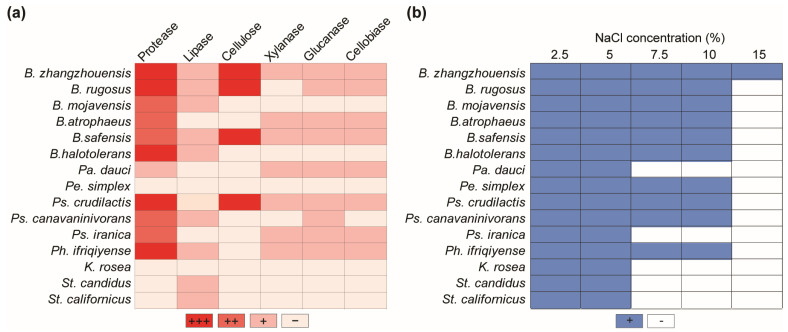
Enzymatic activities of the antagonistic bacteria and their salt tolerance ability. (**a**) Protease, lipase, cellulose, xylanase, glucanase, and cellobiase activities of antagonistic bacteria. ‘+’, ‘++’, and ‘+++’ represent different levels of activity, and ‘−’ indicates an absence of enzymatic activity. (**b**) Salt tolerance of antagonistic bacteria in various concentrations. ‘+’ and ‘−’ represent the presence or lack of bacterial salt tolerance, respectively.

The enzymatic properties of antagonistic bacteria isolated from the plant rhizosphere and phyllosphere, which break down cell wall compounds and degrade lipids and proteins, were determined. *Bacillus* species exhibited stronger enzymatic activity, among others. Specifically, *B. zhangzhouensis* and *B. safensis* demonstrated strong lignocellulolytic, lipolytic, and proteolytic activities. *Bacillus halotolerans* and *B. mojavensis*, on the other hand, were unable to break down plant cell wall components but did exhibit lipase and protease activities. *Bacillus rugosus* was not able to exhibit only xylanase activity, while *B. atrophaeus* and *Pe. dauci* did not produce lipolytic or cellulolytic activities. Most antagonistic bacteria displayed proteolytic activity, except *K. rosea*, *Pe. dauci*, and *Streptomyces* species. *Pseudomonas crudilactis* was shown to be particularly effective in degrading lignocellulosic materials such as cellulose, xylanase, glucanase, and cellobiase. However, it was incapable of decomposing lipids. On the other hand, *Ps. canavaninivorans* showed glucanase, cellobiase, and lipolytic activity, but it did not show any cellulose or xylanase activity. Finally, *Ps. iranica* had no lipolytic or cellulolytic activity, but it did have high xylanase, glucanase, and cellobiase activities. *Pa. dauci* had a similar enzymatic profile. *Streptomyces* species were discovered to be incapable of breaking down plant cell wall components but demonstrated lipase activity. *Phyllobacterium ifriqiyense* exhibited xylanase, glucanase, and cellobiase enzyme activities, as well as high proteolytic activity, but no cellulolytic activity. Finally, the *Pe. simplex* and *K. rosea* species showed no enzymatic activity (Figure 5a). 

### 3.5. Bacterial Growth during Salinity Stress

Various bacterial isolates were examined for their capacity to grow at high sodium chloride concentrations. All antagonistic bacteria were able to thrive in media with NaCl concentrations of up to 5%. All *Bacillus* species were able to tolerate salt up to 10%, but only *B. zhangzhouensis* could thrive in a 15% saline media. *Peribacillus simplex*, *Ps. crudilactis*, *Ps. canavaninivorans*, and *Ph. ifriqiyense* were able to grow in 10% saline media. Meanwhile, *Ps. iranica*, *K. rosea*, *Pa. dauci*, and *Streptomyces* species demonstrated resistance to 5% sodium chloride salinity (Figure 5b).

## 4. Discussion

Antagonistic bacteria play a pivotal role in maintaining plant health by combating soil-borne pathogens. These beneficial bacteria contribute to the overall health of the plant community by acting as natural guardians, providing protection against harmful pathogens [43]. The utilization of antagonistic beneficial bacteria as biological control agents against infectious pathogenic fungi represents one of the most promising and effective strategies in agriculture [44]. Host-associated microorganisms, including bacteria, fungi, and viruses, have evolved mutualistic interactions with their hosts. These relationships are often multifaceted, encompassing nutrient provision, host adaptation to environments, and protection against pathogens [45]. Many bacterial antagonists have evolved sophisticated strategies to protect themselves and their host organisms from potential threats, including competition with other microorganisms and pathogens. Bacterial defense strategies encompass a wide array of mechanisms and molecules, reflecting the diverse ecological niches in which bacteria thrive [46], and are known for their ability to inhibit fungal growth through various modes of action [47,48]. The identification and characterization of bacteria with antagonistic activity against fungal pathogens serve as a valuable resource for the development of environmentally friendly biocontrol agents. Harnessing bacteria with antagonistic activity against fungal pathogens offers a sustainable alternative to chemical fungicides, which can have harmful effects on the environment [49,50,51].

The findings of this study underscore the remarkable adaptability of plant-associated bacteria to thrive in the harsh environmental conditions of the Aral Sea region. The identification of 88 out of 1400 isolates demonstrating antagonistic ability against various fungal pathogens suggests that there is a subset of plant-associated bacteria in the Aral Sea region that possess biocontrol potential. The prevalence of *Bacillus* and *Pseudomonas* species as the most common bacteria with antifungal ability against a wide spectrum of pathogenic fungi is consistent with earlier research that has identified these genera as strong biocontrol agents [52,53,54]. In our investigation, practically all detected *Bacillus* species inhibited fungal pathogens. The most frequent among them was B. zhangzhouensis, a recently identified bacterial species [55]. It was reported that the mass-spectrometric analysis of crude extract of *B. zhangzhouensis* demonstrated phenol, 2, 4-bis(1, 1-dimethyl ethyl) as having active antimicrobial potential against a wide range of the test microorganisms, including Gram-positive and Gram-negative bacteria and fungi [56]. Similarly, *B. rugosus*, *B. safensis*, *B. atrophaeus*, *B. mojavensis*, and *B. halotolerans* were demonstrated to inhibit pathogenic fungi, including *Fusarium* species [57], by producing VOCs and lipopeptides [58,59]. Currently, *B. mojavensis* is utilized as commercial biofungicide against pathogenic fungi [60]. *Pseudomonas* species, the second most diverse antagonistic bacteria in this study, included three species: *Ps. crudilactis*, *Ps. canavaninivorans*, and *Ps. iranica*. *Pseudomonas crudilactis* was the most potent antagonist, inhibiting all pathogenic fungi in vitro. The antimicrobial activity of *Ps. crudilactis*, attributed to the production of antimicrobial lipopeptides, underscores the significance of secondary metabolites in its biocontrol potential [61]. 

*Pa. dauci*, an endophytic actinobacterium isolated from the inner tissue of carrots, was discovered as a producer of potential antimicrobial substances [62]. *Pa. polymyxa* was observed to exhibit strong antifungal activity against *Fusarium graminearum* through the production of antifungal proteins [63,64]. Our results also consistently showed that *Pa. dauci* could suppress practically all *Fusarium* species (four out of six). Similarly, another rhizosphere bacterium, *Pe. ifriqiyense*, also demonstrated antifungal activity against almost all pathogenic fungi, including *Fusarium* species, which was consistent with the study by Kiroiants et al. [65]. 

Two *Streptomyces* spp., *St. candidus* and *St. californicus*, showed varied levels of antifungal ability, and *St. candidus* inhibited a wider range of pathogenic fungi than *St. californicus*. Studies have shown that *Streptomyces* species are known to inhibit fungal growth in vitro [66,67]. The production of several antifungal compounds by *St. candidus*, including lemonomycin, enterocin, pyrazofurin, and avoparcin, underscores its potential as a source of bioactive metabolites for controlling fungal pathogens [68,69,70,71]. Apparently, *St. californicus* also possesses antibacterial and antifungal activities [72,73]. Several species of the *Streptomyces* genus have been commercially developed as biological fungicides due to their ability to produce antimicrobial compounds that inhibit fungal pathogens [74,75,76]. Further studies to characterize the specific antifungal substances produced by promising bacterial isolates are crucial for understanding their biocontrol potential. 

The enzymatic activity analysis revealed the presence of enzymes capable of degrading fungal cell wall components, lipids, and proteins in several bacterial isolates. Enzymes could be integral components of the antagonistic mechanisms employed by bacteria against fungal pathogens [77,78]. The strong lignocellulolytic, lipolytic, and proteolytic activities exhibited by *B. zhangzhouensis* suggest its potential for multifaceted biocontrol and nutrient cycling in the rhizosphere. Understanding the specific enzymes produced by bacteria can serve as a crucial stepping stone for identifying their precise modes of action against fungal pathogens.

The study highlights the salt tolerance of bacterial isolates, particularly *Bacillus*, *Phylobacterium*, and *Pseudomonas* species. The salt tolerance of bacterial isolates, especially in the saline environment of the Aral Sea region [79], is crucial for their survival and functioning in this challenging ecosystem [80,81,82]. The unique ability of *B. zhangzhouensis* to thrive in the highest-salinity medium (15%) underscores its remarkable adaptation to harsh environments. *B. zhangzhouensis* enhances tomato growth by increasing K^+^, Mg^+^, and Ca^2+^ ions while decreasing Na^+^ uptakes [83]. Investigating the underlying mechanisms of salt tolerance in bacteria like *B. zhangzhouensis* can provide valuable insights for engineering stress-tolerant strains with enhanced agricultural applications. The salt-tolerant antagonistic bacterium *B. zhangzhouensis* holds immense potential for the development of biocontrol agents tailored for use in saline agricultural lands [82,84].

Conducting in vivo studies is crucial for evaluating the efficacy of salt-tolerant antagonistic bacteria in controlling fungal diseases in target plants under greenhouse and field conditions. Studying the plant growth promotion potential of salt-tolerant antagonistic bacteria in conjunction with their biocontrol activities offers a comprehensive understanding of their role in plant development and adaptation in harsh environments. The results of the study on plant-associated bacteria in the saline environment of the Aral Sea region hold great significance and make potential contributions to our understanding and applications. This involves examining their ability to control fungal diseases on target plants and evaluating their compatibility with agricultural practices. Overall, this study offers a significant contribution to our understanding of plant-associated bacteria in the Aral Sea region, highlighting their diversity and potential applications in agricultural and environmental contexts. The identification of salt-tolerant bacteria with antagonistic and enzymatic properties holds great promise for the development of environmentally friendly biocontrol agents and the promotion of sustainable agricultural practices in saline environments.

## Figures and Tables

**Table 1:** Collection information for plant samples in Aral Lake, Uzbekistan.

Location	GPS Coordinates	Elevation (m)	Plant Species
Small Aral Sea, western coast	N: 44 502 19 E: 058 207 88	80	*Ferula lehmannii*, *Amberboa turanica*, *Zygophyllum atriplicoides*
Small Aral Sea, western coast	N: 44 503 36 E: 058 20 900	42	*Rheum turkestanicum*, *Zygophyllum sp.*, *Lactuca serriola*
Small Aral Sea, western coast	N: 44 50 335 E: 058 20 978	46	*Ferula* sp., *Zygophyllum oxianum*, *Senecio subdentatus*
Small Aral Sea, western coast	N: 44 503 40 E: 058 212 59	40	*Artemisia* sp., *Jurinea* sp.
Small Aral Sea, western coast	N: 44 50 361 E: 058 21 525	39	*Astragalus villosissimus*, *Datura* sp.
Small Aral Sea, western coast	N: 44 505 39 E: 058 230 98	22	*Kalidium foliatum*
Small Aral Sea, southwestern coast	N: 44 38 058 E: 058 281 58	34	*Tamarix* sp., *Euphorbia inderiensis*, *Euphorbia* sp., *Anabasis salsa*, *Eremopyrum orientale*, *Hyoscyamus pusillus*
Dry seabed	N: 44 155 22 E: 058 515 24	34	*Tamarix* sp.
Muynak, origin sea ex-coast	N: 43 78 967 E: 059 03 398	46	*Chamaesphacos ilicifolius*
Muynak, origin sea ex-coast	N: 43 79 009 E: 059 03 610	46	*Stipagrostis karelinii*, *Heliotropium ellipticum*, *Halocharis hispida*, *Corispermum lehmannianum*, *Salsola* sp.

**Table 2:** Molecular identification of antagonistic bacteria by 16S RNA sequence analysis and their taxonomic status.

Phylum	Class	Order	Family	Genus/Species	Number of Isolates
Bacillota	Bacilli	Bacillales	Bacillaceae	*Bacillus zhangzhouensis*	47
				*B. rugosus*	15
				*B. mojavensis*	2
				*B. atrophaeus*	7
				*B. safensis*	1
				*B. halotolerans*	1
			Paenibacillaceae	*Paenibacillus dauci*	1
				*Peribacillus simplex*	1
Proteobacteria	γ-Proteobacteria	Pseudomonadales	Pseudomonadaceae	*Pseudomonas crudilactis*	5
				*Ps. canavaninivorans*	1
				*Ps. iranica*	1
	α-Proteobacteria	Hyphomicrobiales	Phyllobacteriaceae	*Phyllobacterium ifriqiyense*	2
Actinomycetota	Actinomycetes	Micrococcales	Micrococcaceae	*Kocuria rosea*	1
		Kitasatosporales	Streptomycetaceae	*Streptomyces candidus*	2
				*St. californicus*	1

**Table 3:** Distribution of antagonistic species in rhizosphere and phyllosphere parts of plants.

Family	Species	Bacterial Antagonists	Root	Twig
Apiaceae	*Ferula lehannii*	*Pa. dauci*	-	1
		*St. californicus*	-	1
	*Ferula* sp.	*B. mojavensis*	1	-
Asteraceae	*Amberboa turanica*	*K. rosea*	1	-
		*St. candidus*	1	-
		*B. atrophaeus*	-	1
	*Lactuca serriola*	*B. rugosus*	1	-
		*B. atrophaeus*	1	-
	*Senecio subdentatus*	*B. zhangzhouensis*	5	-
		*B. rugosus*	7	-
	*Artemisia* sp.	*-*	-	-
	*Jurinea* sp.	*-*	-	-
Boraginaceae	*Heliotropium ellipticum*	*-*	-	-
Chenopodiaceae	*Halocharis hispida*	*B. atrophaeus*	-	3
		*B. rugosus*	-	2
	*Corispermum lehmannianum*	*Ps. crudilactis*	-	1
		*Ps. canavaninivorans*	1	-
	*Salsola* sp.	*-*	-	-
	*Anabasis salsa*	*B. zhangzhouensis*	-	1
		*B. rugosus*	2	-
	*Kalidium foliatum*	*B. zhangzhouensis*	14	-
Euphorbiaceae	*Euphorbia* sp.	*-*	-	-
	*Euphorbia inderiensis*	*B. rugosus*	1	1
		*Pe. simplex*	1	-
Fabaceae	*Astragalus villosissimus*	*Ps. crudilactis*	-	1
		*B. zhangzhouensis*	-	19
Lamiaceae	*Chamaesphacos ilicifolius*	*Ps. iranica*	1	-
Poaceae	*Stipagrostis karelinii*	*Ps. crudilactis*	2	-
	*Eremopyrum orientale*	*B. safensis*	-	1
		*B. zhangzhouensis*	-	2
Polygonaceae	*Rheum turkestanicum*	*-*	-	-
Solanaceae	*Hyoscyamus pusillus*	*St. candidus*	-	1
	*Datura* sp.	*Ps. crudilactis*	-	1
Tamaricaceae	*Tamarix* sp.	*-*	-	-
		*B. halotolerans*	-	1
Zygophyllaceae	*Zygophyllum atriplicoides*	*B. atrophaeus*	2	-
	*Zygophyllum* sp.	*B. rugosus*	-	1
		*B. mojavensis*	-	1
		*B. zhangzhouensis*	-	1
	*Zygophyllum oxianum*	*B. zhangzhouensis*	5	-
		*Ph. ifriqiyense*	2	-

## Data Availability

The authors declare that the experimental data published in this paper will be made accessible upon request for interested readers. All 16S rRNA gene sequences of the new strains can be found under accession numbers: PP267998.1 (*B. zhangzhouensis* IGPEB-AS-01), IGPEB-AS-02 PP267999.1 (*B. rugosus*), PP268000.1 (*B. mojavensis* IGPEB-AS-03), PP268001.1 (*B. atrophaeus* IGPEB-AS-04), PP268002.1 (*B. safensis* IGPEB-AS-05), PP268003.1 (*B. halotolerans* IGPEB-AS-06), PP268004.1 (*Pa. dauci* IGPEB-AS-07), PP268005.1 (*Pe. simplex* IGPEB-AS-08), PP268006.1 (*Ps. crudilactis* IGPEB-AS-09), PP268007.1 (*Ps. canavaninivorans* IGPEB-AS-10), PP268008.1 (*Ps. iranica* IGPEB-AS-11), PP268009.1 (*Ph. ifriqiyense* IGPEB-AS-12), PP268010.1 (*K. rosea* IGPEB-AS-13), PP268011.1 (*St. candidus* IGPEB-AS-14), PP268012.1 (*St. californicus* IGPEB-AS-15). All data generated or analyzed during this study are included in this published article.
